# Attempt to Isolate Elephant Endotheliotropic Herpesvirus (EEHV) Using a Continuous Cell Culture System

**DOI:** 10.3390/ani10122328

**Published:** 2020-12-07

**Authors:** Kornravee Photichai, Thunyamas Guntawang, Tidaratt Sittisak, Varankpicha Kochagul, Phongsakorn Chuammitri, Chatchote Thitaram, Hathairat Thananchai, Teera Chewonarin, Korawan Sringarm, Kidsadagon Pringproa

**Affiliations:** 1 Department of Veterinary Biosciences and Veterinary Public Health, Faculty of Veterinary Medicine, Chiang Mai University, Chiang Mai 50100, Thailand; kornravee_ph@cmu.ac.th (K.P.); thunyamas_g@cmu.ac.th (T.G.); Tidaratt_s@cmu.ac.th (T.S.); phongsakorn.c@cmu.ac.th (P.C.); 2 Veterinary Diagnostic Laboratory, Faculty of Veterinary Medicine, Chiang Mai University, Chiang Mai 50100, Thailand; chomchay.k@cmu.ac.th; 3 Department of Companion Animals and Wildlife Clinics, Faculty of Veterinary Medicine, Chiang Mai University, Chiang Mai 50100, Thailand; chatchote.thitaram@cmu.ac.th; 4 Department of Microbiology, Faculty of Medicine, Chiang Mai University, Chiang Mai 50200, Thailand; hathairat.t@cmu.ac.th; 5 Department of Biochemistry, Faculty of Medicine, Chiang Mai University, Chiang Mai 50200, Thailand; teera.c@cmu.ac.th; 6 Department of Animal and Aquatic Sciences, Faculty of Agriculture, Chiang Mai University, Chiang Mai 50200, Thailand; korawan.s@cmu.ac.th; 7 Excellence Center in Veterinary Bioscience, Chiang Mai University, Chiang Mai 50100, Thailand

**Keywords:** elephant endotheliotropic herpesvirus, isolation, cell culture, in vitro

## Abstract

**Simple Summary:**

Elephant endotheliotropic herpesvirus-hemorrhagic disease (EEHV-HD) is one of the most important viral infectious diseases in young Asian elephants (*Elephas maximus*). To date, in vitro isolation or propagation of EEHV has so far unsuccessful. Findings in the present study suggest that the U937 cells, a cell line derived from the human myeloid leukemia patient, can be used to isolate and propagate EEHV in vitro. Replication of EEHV in the U937 cells is determined by the presence of EEHV DNA polymerase antigens in the infected cells. However, the replication in these cells was shown to be restricted and observed only in the early passages of virus infection. Although EEHV replication in U937 cells has only occurred in the early passages, our findings have shed some light on the feasibility of using this cell line for further in vitro EEHV isolation.

**Abstract:**

Elephant endotheliotropic herpesvirus (EEHV) infection is known to cause acute fatal hemorrhagic disease, which has killed many young Asian elephants (*Elephas maximus*). Until recently, in vitro isolation and propagation of the virus have not been successful. This study aimed to isolate and propagate EEHV using continuous cell lines derived from human and/or animal origins. Human cell lines, including EA. hy926, A549, U937, RKO, SW620, HCT-116 and HT-29, and animal cell lines, including CT26.CL25 and sp2/0-Ag14, were investigated in this study. Mixed frozen tissue samples of the heart, lung, liver, spleen and kidney obtained from fatal EEHV1A- or EEHV4-infected cases were homogenized and used for cell inoculation. At 6, 24, 48 and 72 h post infection (hpi), EEHV-inoculated cells were observed for cytopathic effects (CPEs) or were assessed for EEHV infection by immunoperoxidase monolayer assay (IPMA) or quantitative PCR. The results were then compared to those of the mock-infected controls. Replication of EEHV in the tested cells was further determined by immunohistochemistry of cell pellets using anti-EEHV DNA polymerase antibodies or re-inoculated cells with supernatants obtained from passages 2 or 3 of the culture medium. The results reveal that no CPEs were observed in the tested cells, while immunolabeling for EEHV gB was observed in only U937 human myeloid leukemia cells. However, quantitation values of the EEHV terminase gene, as well as those of the EEHV gB or EEHV DNA polymerase proteins in U937 cells, gradually declined from passage 1 to passage 3. The findings of this study indicate that despite poor adaptation in U937 cells, this cell line displays promise and potential to be used for the isolation of EEHV1 and EEHV4 in vitro.

## 1. Introduction

Elephant endotheliotropic herpesvirus (EEHV) is an enveloped, linear dsDNA virus classified in the subfamily Betaherpesvirinae, genus *Proboscivirus*. This virus has so far been divided into 8 different genotypes including EEHV1A, EEHV1B and EEHV2 to EEHV7 [1,2]. According to the findings of previous reports, EEHV1 and EEHV4 are known to be the most common causes of acute fatal hemorrhagic disease especially in young to juvenile Asian elephants (*Elephas maximus*) [1,3,4]. It has been postulated that mucosal secretion from asymptomatic elephants to one another is a major route of transmission [5,6,7]. Ulceration at the oral cavity and laryngeal areas, along with massive hemorrhaging and edema of the internal organs and subcutaneous tissues, are some of the commonly associated pathological outcomes of this disease [3,4,6]. Treatment of EEHV-infected calves with antiviral medication, such as famciclovir or acyclovir, at the early stages of infection along with supportive therapy, has been reported to be successful in only some cases [3,8,9,10]. Currently, there are no available vaccines, specific preventive protocols or alternative treatments that have been found to be effective against this disease. This could be due to the fact that the isolation and propagation of EEHV were not found to be successful in vitro.

Virus isolation is recognized as just one of the “gold standard” methods that are used to diagnose several types of viruses, for which alternative diagnostic methods must be compared [11,12,13,14]. In pharmacological studies, direct antiviral drug testing requires a laboratory cell culture model for the growth and propagation of the virus [15,16]. The cell culture model is needed for the study of virus-cell interaction and host cellular response research [17,18]. However, despite the fact that the common method for virus isolation is cell culture inoculation [19], previous studies have shown that the culturing of EEHV has been unsuccessful in several cell lines or primary cells including those of the African green monkey kidney (Vero and MARC-145 (Meat Animal Research Center-145)), baby hamster kidney-21 (BHK-21), rabbit kidney-13 (RK-13), Mardin–Darby canine kidney (MDCK), Mardin–Darby bovine kidney (MDBK), human rectal tumor (HrT-18G), equine endothelial cells, equine dermal cells, embryonated chicken fibroblasts, primary bovine embryo lung cells and human foreskin fibroblasts [1,4,20,21]. However, although a recent study has demonstrated that EEHV could be cultivated in vitro using elephant fibroblast (ENL-2) cells, the virus was unable to be propagated beyond passage 3 [20]. These findings have brought attention to the feasibility of the hypothesis that the isolation of EEHV may be achieved in vitro using susceptible cell culture models.

Similar to other herpesviruses, it has been speculated that EEHV is able to evade host immune responses and become persistent in infected elephants [22]. However, the cell types that are targeted by the virus during persistent infections remain to be determined [22]. Interestingly, our recent report has shown that monocytes/macrophages, endothelia and epithelia of the alimentary tracts of elephants are targeted and serve as the predominant cell types that are favored for EEHV infection and replication during the acute phase of infection [23]. This finding brings promise to the speculation that these cell types may be used for in vitro EEHV isolation. Thus, the present study aimed to isolate and propagate EEHV using various cell lines originating from the epithelia of the intestines, as well as by using the endothelia and monocytic lineage cells of humans or murine.

## 2. Materials and Methods

### 2.1. Preparation of EEHV1A and EEHV4 Inoculum

Preparation of the EEHV inoculums was accomplished by following the method previously described [20] with slight modifications. Briefly, mixed frozen tissue samples (taken from the lungs, liver, heart, spleen and kidneys) of elephant calves that died due to EEHV1A or EEHV4 infection in the year 2018 were obtained and used in the present study. Tissue samples were chopped into small pieces and then homogenized in Roswell Park Memorial Institute medium, (RPMI)-1640 medium, along with a homogenizer. They were then centrifuged at 2090× *g* for 10 min at 4 °C. The supernatant was collected and then filtered through a 0.22 µm filter membrane. Filtrated fluid was then collected as the inoculum and kept at −20 °C for use in further studies.

### 2.2. Cell Lines and Culture Media

A total of 9 cell lines were used in this study. These were comprised of the human umbilical cord endothelial cell line (EA.hy926), human lung carcinoma cell line (A549), human myeloid leukemia cell line (U937), human colon carcinoma cell line (RKO), human colon carcinoma from lymph node metastatic sites cell line (SW620), human colorectal carcinoma cell line (HCT116), human colorectal adenocarcinoma cell line (HT-29), mouse colon carcinoma cell line (CT26.CL25) and mouse myeloma cell line (Sp2/0-Ag14, all obtained from the American Type Culture Collection (ATCC), Manassas, VA, USA). Culture media for the U937 and CT26.CL25 cells were comprised of RPMI-1640 medium, while media for the other cell lines were comprised of Dulbecco’s Modified Eagle’s Medium (DMEM). Media were supplemented with 10% (*v*/*v*) fetal bovine serum (FBS) and 1% antimicrobial (10,000 U/mL of Penicillin G, 10,000 µg/mL of Streptomycin and 25 µg/mL of Amphotericin B, obtained from Carlsbad, CA, USA) and then used as complete media. Cells were maintained under standard culture conditions comprised of 37 °C and 5% CO_2_.

### 2.3. Cell Inoculation

Cells were grown in 96- and 24-well microtiter plates until they reached 70% confluency. Inoculation was done in each well with prepared EEHV1A or EEHV4 inoculums for 60 min at room temperature (RT) as has been previously described [24]. Briefly, 100 µL or 300 µL of the inoculum was added to the 96- or 24-well microtiter plates, respectively. After 1 h, supernatants were discarded, replaced with complete medium and maintained at 37 °C in 5% CO_2_. The medium alone was used instead of the inoculums to serve as the mock-infected control. Cytopathic effects (CPEs) of the EEHV-inoculated cells were observed daily under an inverted light microscope and then compared to the mock-infected controls. At 24, 48 and 72 h post inoculation (hpi), the culture medium was collected. Subsequently, cells in 96-well plates were fixed with 4% buffered formalin at RT for 15 min, while cells in 24-well plates were collected as cell pellets and fixed with formalin. Media were quantified for the EEHV genome using qPCR, while fixed cells were processed for analyzing EEHV infection by immunoperoxidase monolayer assay (IPMA), immunofluorescence or immunohistochemistry, as described below. Cell lines that supported EEHV infection were then used to create EEHV virions by serial passaging, as described below.

### 2.4. Viral Serial Passaging

To obtain progeny viruses from the EEHV-inoculated cells, serial passage of EEHV was made on the monolayer of the selected continuous cell line in a T25 flask, as previously described [25]. Briefly, supernatants at day 3 of the EEHV1A- or EEHV4-inoculated cells were collected, stored at −80 °C and labeled as viral passage 1. To create viral passage 2, 1 mL of the EEHV passage 1 was then used to inoculate a T25 flask of the fresh cells that were used to inoculate viral passage 1, as described earlier. Subsequently, the supernatant was collected on day 3 after inoculation and labeled as viral passage 2, which was then used to create viral passage 3, respectively. To determine and compare the degree of infectivity among EEHV passages 1, 2 and 3, they were used to inoculate selected cells which were then harvested on day 3 of the post-inoculation period. These cells were centrifuged at 2090× *g* for 10 min to collect cell pellets and were then fixed with 10% buffered formalin. EEHV infection in these cells was determined by immunohistochemistry using the polyclonal antibody against the EEHV DNA polymerase (DNAPol), as described below.

### 2.5. Quantitative PCR

The supernatant of EEHV1A-inoculated, EEHV4-inoculated and mock-infected controls obtained from each time point of viral passage 1 were subjected to DNA extraction using NucleoSpin DNA II Kits (Macherey-Nagel GmbH, Duren, Germany) according to the manufacturer’s instructions. Viral terminase-specific primers were used to quantify the number of viral copies obtained from the extracted DNA when compared with the standard curved, as has been previously described [26]. PCR was performed using a SensiFast SYBR^®^Hi-ROX kit (Bioline, Luckenwalde, Germany) coupled with an ABI7300 thermocycler (Applied Biosystems, Foster, CA, USA). The absolute quantitative values were calculated based on the threshold cycles (Ct) of the terminase genes that were obtained from the extracted DNA samples. These values were then compared to the known standard DNA template and presented as viral genome copies (vgc)/mL as has been previously described [27]. Experiments were done in triplicate, and all data were obtained and analyzed as described below.

### 2.6. Immunoperoxidase Monolayer Assay (IPMA)

IPMA of EEHV-inoculated cells was performed in 96-well plates as has been previously described [28]. Briefly, after cells were fixed with 4% formalin for 15 min at RT, they were washed 3 times with 0.25% (*v*/*v*) Triton X-100 in PBS (0.25% PBST) for 5 min each. Thereafter, cells were incubated with 1% (*w*/*v*) bovine serum albumin (BSA) for 15 min, and the washing steps were then repeated prior to incubation with primary rabbit anti-EEHV gB (1:500) antibody [6] for 1 h at RT. After the antibody solution was removed, cells were washed with PBS and incubated with normal goat serum (1:5 in PBS) for 30 min at RT. Secondary HRP conjugated goat anti-rabbit antibody (1:200) was then applied for 1 h at RT, followed by the 3,3′-diaminobenzidine (DAB) substrate in order to develop the relevant signals. Plates were then dried, and photos were taken under an inverted light microscope.

### 2.7. Immunofluorescence

Immunofluorescent staining of the EEHV-inoculated cells was done using the method previously described [6]. Briefly, the cultures were fixed with 4% (*v*/*v*) paraformaldehyde for 15 min at RT and treated with 0.25% (*v*/*v*) Triton X-100 in PBS (0.25% PBST) for 15 min. The cells were then incubated with 1% (*w*/*v*) bovine serum albumin (BSA) in PBST for 30 min at RT, followed by incubation with specific primary antibodies diluted with 1% (*w*/*v*) BSA in 0.25% PBST at 37 °C for 2 h. The primary antibody was rabbit polyclonal anti-EEHV gB (1:500; [6]). The secondary antibody was incubated for 45 min at RT with FITC-conjugated goat anti-rabbit antibodies (Jackson ImmunoResearch, Suffolk, UK) at a dilution of 1:200. The nuclei were counterstained using bisbenzimide (Sigma-Aldrich, Merck, Darmstadt, Germany) for 10 min at RT. The cultures were then analyzed, and photos were taken under an inverted fluorescent microscope.

### 2.8. Immunohistochemistry (IHC)

To determine the replication of EEHV in selected cells, cells were infected with supernatants obtained from passages 1, 2 or 3 of the EEHV-inoculated cells. The cells were then harvested in 1.5 mL tubes and centrifuged at 2090× *g* for 5 min to collect cell pellets. Thereafter, cells were fixed with 4% formalin, processed for paraffin-embedded tissue and subjected to immunohistochemistry as has been previously described [6,23]. The primary antibody was rabbit polyclonal anti-EEHV DNA polymerase (1:800 in PBS; [23]). Normal rabbit serum was used instead of the primary antibody and served as the negative control. Immunolabeling positive cells were examined using a light microscope.

### 2.9. Data Analysis

All data were analyzed and presented in a descriptive analysis using GraphPad Prism 5 (GraphPad Inc., La Jolla, CA, USA).

## 3. Results

### 3.1. Cytopathic Effects (CPEs) of EEHV-Inoculated Cells

**Figure 1:**
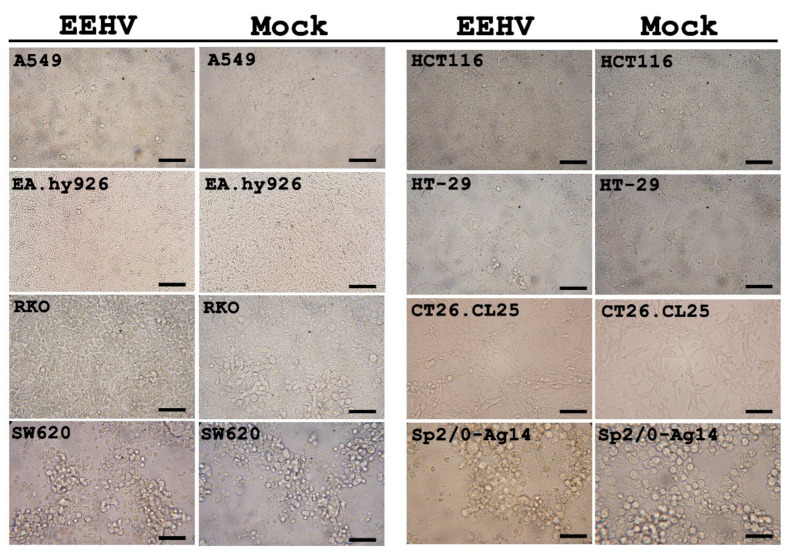
Cell morphology of the A549, HCT116, EA.hy926, HT-29, RKO, CT26.CL25, SW620 and Sp2/0-Ag-14 cells after being inoculated with the EEHV inoculum. At 72 hpi, there were no obvious cytological changes to the EEHV-inoculated cells when compared with the mock-infected control. Scale bars ~200 µm. EEHV: elephant endotheliotropic herpesvirus.

**Figure 2:**
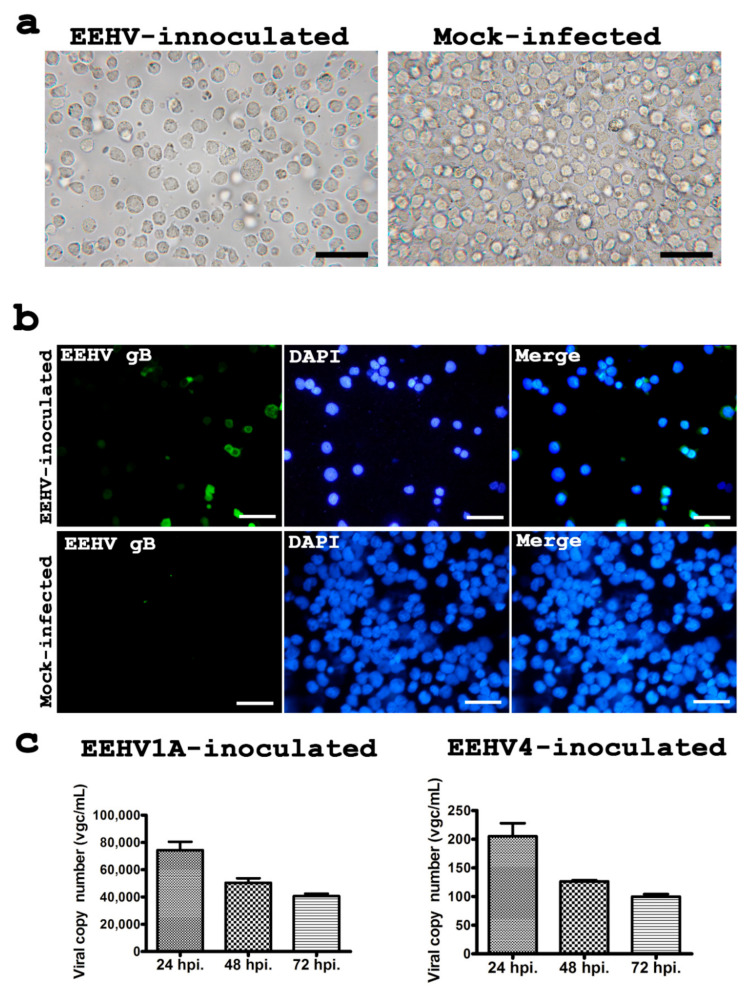
Cell morphology, immunolabeling for EEHV gB and determination of EEHV terminase genes in the U937 cells after inoculation with EEHV. At 72 hpi, although no cytological changes were observed in the U937 cells (**a**), immunolabeling for the EEHV gB was shown to be positive by immunofluorescence in the EEHV-inoculated group (**b**). Quantitative PCR presented as viral genome copies (vgc/mL) of the U937 cell culture supernatant at 24, 48 and 72 hpi indicated that there was a reduction of EEHV in both the EEHV1A-inoculated and EEHV4-inoculated cells (**c**). Scale bars in (**a**) ~200 µm, in (**b**) ~300 µm.

At 24, 48 and 72 hpi, there were no obvious CPEs in the EEHV-inoculated cells when compared to the mock-infected controls (Figure 1). EEHV gB immunolabeling was also not detected in A549, HCT116, EA.hy926, HT-29, RKO, CT26.CL25, SW620 and Sp2/0-Ag-14 cells (data not shown). Even though CPEs were not seen in the U937 cells (Figure 2a), the EEHV gB antigen was detected in U937 cells in both EEHV1A-inoculated and EEHV4-inoculated cells by immunofluorescense (Figure 2b). Expression of EEHV gB was observed in the cytoplasm of U937 cells at up to 60% of the inoculated cells at 72 hpi (Figure 2b). These results indicate that EEHV1A and EEHV4 were tethered to the U937 cells.

### 3.2. Quantification of EEHV Genome in U937 Cell Supernatant

To quantify the EEHV genome in the U937 culture media at each time point, supernatants were collected on days 1, 2 and 3 after inoculation. The absolute number of EEHV terminase genes in the supernatants was then determined using qPCR. As is shown in Figure 2c, the copied numbers of the EEHV terminase genes in the supernatant gradually declined on days 2 and 3 post inoculation when compared to day 1. These results indicate that EEHV can enter U937 cells; however, the infection was restricted which then resulted in a decline of the viral genome in the culture supernatant.

### 3.3. Detection of EEHV Replication in U937 Cells

**Figure 3:**
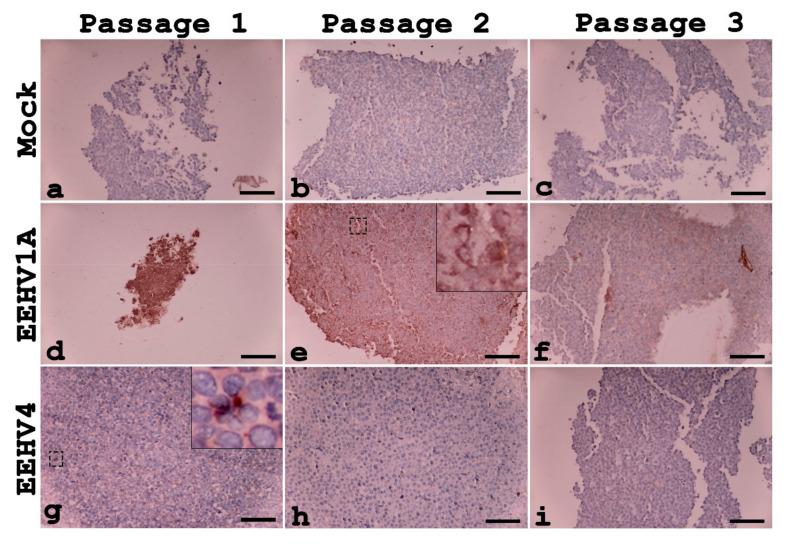
Representative photomicrographs of U937 cell pellets inoculated with supernatant obtained from passages 1, 2 and 3 of the EEHV-inoculated U937 cells. Immunolabeling for EEHV DNA polymerase antibodies was found to be strongly positive in the cytoplasm (inset) of the EEHV1A-inoculated (**d**–**f**) and EEHV4-inoculated (**g**–**i**) cells when compared to the mock-infected controls (**a**–**c**). However, immunolabeling in the positive cells for EEHV DNAPol declined in passages 3 and 2 of the EEHV1A- and EEHV4-inoculated groups, respectively. Scale bars ~800 µm.

To determine whether U937 cells support the infection and replication of EEHV, supernatant from passages 1, 2 or 3 was inoculated onto naïve U937 cells. Cells were then collected and analyzed for the presence of EEHV DNA polymerase non-structural proteins. As compared to the mock-infected control (Figure 3a–c), antigens of EEHV DNA polymerase were observed in the U937 cells when they were inoculated with the EEHV1A or EEHV4 inoculums (Figure 3d–i) indicating viral replication in these cells. Unfortunately, positive signals of viral DNA polymerase gradually declined in the U937 cells that were infected with passage 2 of EEHV1A (Figure 3d–f) and passage 1 of EEHV4 (Figure 3g–i). In passage 3, immunolabeling for EEHV DNA polymerase in the EEHV4-inoculated U937 cells was undetected (Figure 3i). These results indicate that U937 can support EEHV1A and EEHV4 replication only in their early passages.

## 4. Discussion

Virus isolation using the cell culture system has been extensively used as a standard laboratory procedure for the propagation of viruses in vitro [12,15,16] (Kabelo et al., 2020; Meister et al., 2019; Taylor, 2013). This procedure has not only produced progeny viruses, but it can also be used to study in vitro viral pathogenesis in the pursuit of alternative therapeutic medications or for the control of these viruses [12,15,17,18] (Kabelo et al., 2020; Meister et al., 2019; Pringproa et al., 2015; Wu et al., 2017). In EEHV, however, isolation and propagation of viruses using the cell culture system have not been successful thus far. Interestingly, our recent report has demonstrated that endothelial cells, bone marrow cells, monocytes/macrophages, salivary gland cells and epithelia of the intestinal tracts are favored for EEHV1A and EEHV4 replication in vivo [23] (Guntawang et al., 2020). These findings bring attention to the fact that these cell types may be used as target cells for the propagation of EEHV in vitro. Furthermore, since betaherpesviruses, such as human cytomegalovirus (HCMV), have been shown to efficiently infect and be replicated in the adenocarcinoma lung epithelial cell line [29] (Vitenshtein et al., 2016), we then recruited a range of the cell line that was derived from endothelial, monocyte/macrophages, bone marrow and the epithelia of the alimentary tract and lungs for a closer investigation of EEHV infection in this study.

Findings in the present study indicating that EEHV DNA polymerase antigens were present in U937 cells strongly suggest that this cell line is favored for in vitro EEHV infection and replication. The U937 cell is a pro-monocytic cell line that is derived from human myeloid leukemia patients [30] (Stockbauer et al., 1983). This cell line can be differentiated into macrophages and dendritic cells [30,31,32] (Chanput et al., 2015; Kabel et al., 1983; Stockbauer et al., 1983). Previous studies have shown that U937 cells can act as a potential target of several viruses, such as herpes simplex virus type 1 (HSV-1), human immunodeficiency virus (HIV) and Dengue virus (DENV) [33,34,35] (Moriuchi et al., 1998; Puerta-Guardo et al., 2010; Tenney and Morahan, 1987). Depending on viral species, infection in U937 cells requires different cellular receptors for viral attachment and entry. In betaherpesviruses, such as HCMV, infection and replication of viruses in host cells require specific receptors including platelet-derived growth factors (PDGFs) and integrins [36,37,38,39] (Kabanova et al., 2016; Sinzger and Jahn, 1996; Soroceanu et al., 2008; Wang et al., 2005). Although it remains unknown what receptors in elephant cells are required for EEHV infection, the fact that PDGFs are ubiquitous and expressed in U937 cells [40,41] (de Bruin et al., 2004; Savikko and von Willebrand, 2001) has brought this cell line significant attention as a promising tool for in vitro EEHV cultivation. However, it remains to be determined why EEHV infection in U937 cells was restricted and why it was only supported in the early passages of virus infection.

It has been shown that cell lines derived from endothelia, as well as intestinal and lung epithelia, failed to propagate EEHV in vitro [1,4,20] (Ossent et al., 1990; Pavulraj et al., 2019; Richman et al., 1999). However, infection and propagation of EEHV in the cell lines, such as that of the ENL-2 cells, has been recently demonstrated despite the fact that it remains to be determined why infection is restricted and occurred only in the early passages [20] (Pavulraj et al., 2019). In view of the recent findings, it also remains unclear why the EEHV progeny virus was declined in passages 2 and 3 and did not produce productive infection in U937 cells. Several possibilities may account for why this occurred. One possibility is that during the growth process in living cells, the virus requires the proteins of host cells for replication. The specific proteins in elephant host cells may also be necessary for the replication of EEHV1A and EEHV4. Moreover, the fulfillment of the adaptation of EEHVs in human host cells may have been inhibited by interspecies differences. To analyze and explain these hypotheses, further studies are required. In summary, although EEHV internalization and replication in U937 cells only occurred in early passages, the findings of this study shed some light on the feasibility of using this cell line for further in vitro EEHV isolation.

## Abbreviations

ATCC	American Type Culture Collection
BHK-21	Baby hamster kidney-21
BSA	Bovine serum albumin
CPEs	Cytopathic effects
DAB	3,3′-diaminobenzidine
DENV	Dengue virus
DMEM	Dulbecco’s Modified Eagle’s Medium
DNAPol	DNA polymerase
EEHV	Elephant endotheliotropic herpesvirus
FBS	Fetal bovine serum
FITC	Fluorescein isothiocyanate
HIV	Human immunodeficiency virus
HPI	Hours post infection
HrT-18G	Human rectal tumor
HSV-1	Herpes simplex virus type 1
IHC	Immunohistochemistry
IFA	Immunofluorescence assay
IPMA	Immunoperoxidase monolayer assay
MARC-145	Meat Animal Research Center-145
MDBK	Mardin–Darby bovine kidney
MDCK	Mardin–Darby canine kidney
PBS	Phosphate-buffered saline
PBST	Triton X-100 in PBS
PDGFs	Platelet-derived growth factors
RK-13	Rabbit kidney-13 (RK-13)
RPMI	Roswell Park Memorial Institute medium
RT	Room temperature

## References

[1] Richman L.K., Montali R.J., Garber R.L., Kennedy M.A., Lehnhardt J., Hildebrandt T., Schmitt D., Hardy D., Alcendor D.J., Hayward G.S. (1999). Novel endotheliotropic herpesviruses fatal for Asian and African elephants. Science.

[2] Wilkie G.S., Davison A.J., Kerr K., Stidworthy M.F., Redrobe S., Steinbach F., Dastjerdi A., Denk D. (2014). First fatality associated with elephant endotheliotropic herpesvirus 5 in an Asian elephant: Pathological findings and complete viral genome sequence. Sci. Rep..

[3] Ling P.D., Long S.Y., Fuery A., Peng R.-S., Heaggans S.Y., Qin X., Worley K.C., Dugan S., Hayward G.S. (2016). Complete genome sequence of elephant endotheliotropic herpesvirus 4, the first example of a GC-rich branch proboscivirus. MSphere.

[4] Ossent P., Guscetti F., Metzler A., Lang E., Rübel A., Hauser B. (1990). Acute and fatal herpesvirus infection in a young Asian elephant (*Elephas maximus*). Vet. Pathol..

[5] Garner M., Helmick K., Ochsenreiter J., Richman L.K., Latimer E., Wise A., Maes R., Kiupel M., Nordhausen R., Zong J. (2009). Clinico-pathologic features of fatal disease attributed to new variants of endotheliotropic herpesviruses in two Asian elephants (*Elephas maximus*). Vet. Pathol..

[6] Kochagul V., Srivorakul S., Boonsri K., Somgird C., Sthitmatee N., Thitaram C., Pringproa K. (2018). Production of antibody against elephant endotheliotropic herpesvirus (EEHV) unveils tissue tropisms and routes of viral transmission in EEHV-infected Asian elephants. Sci. Rep..

[7] Latimer E., Zong J.-C., Heaggans S.Y., Richman L.K., Hayward G.S. (2011). Detection and evaluation of novel herpesviruses in routine and pathological samples from Asian and African elephants: Identification of two new probosciviruses (EEHV5 and EEHV6) and two new gammaherpesviruses (EGHV3B and EGHV5). Vet. Microbiol..

[8] Dastjerdi A., Seilern-Moy K., Darpel K., Steinbach F., Molenaar F. (2016). Surviving and fatal elephant endotheliotropic herpesvirus-1A infections in juvenile Asian elephants–lessons learned and recommendations on anti-herpesviral therapy. Bmc Vet. Res..

[9] Schmitt D.L., Hardy D.A., Montali R.J., Richman L.K., Lindsay W.A., Isaza R., West G. (2000). Use of famciclovir for the treatment of endotheliotrophic herpesvirus infections in Asian elephants (*Elephas maximus*). J. Zoo Wildl. Med..

[10] Sripiboon S., Angkawanish T., Boonprasert K., Sombutputorn P., Langkaphin W., Ditcham W., Warren K. (2017). Successful treatment of a clinical elephant endotheliotropic herpesvirus infection: The dynamics of viral load, genotype analysis, and treatment with acyclovir. J. Zoo Wildl. Med..

[11] Bello M.B., Yusoff K., Ideris A., Hair-Bejo M., Peeters B.P., Omar A.R. (2018). Diagnostic and vaccination approaches for newcastle disease virus in poultry: The current and emerging perspectives. Biomed. Res. Int..

[12] Kabelo T., Fana E., Lebani K. (2020). Assessment of the sensitivity of primary cells and cell lines to the Southern African Territories (SAT) serotypes in the diagnosis of foot-and-mouth disease virus. Heliyon.

[13] Lin P., Wang H., Cheng Y., Song S., Sun Y., Zhang M., Guo L., Yi L., Tong M., Cao Z. (2018). Loop-mediated isothermal amplification-single nucleotide polymorphism analysis for detection and differentiation of wild-type and vaccine strains of mink enteritis virus. Sci. Rep..

[14] Peletto S., Caruso C., Cerutti F., Modesto P., Biolatti C., Pautasso A., Grattarola C., Giorda F., Mazzariol S., Mignone W. (2018). Efficient isolation on Vero. DogSLAMtag cells and full genome characterization of Dolphin Morbillivirus (DMV) by next generation sequencing. Sci. Rep..

[15] Meister T.L., Bruening J., Todt D., Steinmann E. (2019). Cell culture systems for the study of hepatitis E virus. Antivir. Res..

[16] Taylor D.R. (2013). Evolution of cell culture systems for HCV. Antivir. Ther..

[17] Pringproa K., Rungsiwiwut R., Tantilertcharoen R., Praphet R., Pruksananonda K., Baumgärtner W., Thanawongnuwech R. (2015). Tropism and induction of cytokines in human embryonic-stem cells-derived neural progenitors upon inoculation with highly-pathogenic avian H5N1 influenza virus. PLoS ONE.

[18] Wu Y., Prager A., Boos S., Resch M., Brizic I., Mach M., Wildner S., Scrivano L., Adler B. (2017). Human cytomegalovirus glycoprotein complex gH/gL/gO uses PDGFR-α as a key for entry. Plos Pathog..

[19] Hodinka R.L., Kaiser L. (2013). Point-counterpoint: Is the era of viral culture over in the clinical microbiology laboratory?. J. Clin. Microbiol..

[20] Pavulraj S., Eschke K., Prahl A., Flügger M., Trimpert J., van den Doel P.B., Andreotti S., Kaessmeyer S., Osterrieder N., Azab W. (2019). Fatal elephant endotheliotropic herpesvirus infection of two young Asian elephants. Microorganisms.

[21] Seilern-Moy K., Darpel K., Steinbach F., Dastjerdi A. (2016). Distribution and load of elephant endotheliotropic herpesviruses in tissues from associated fatalities of Asian elephants. Virus Res..

[22] Srivorakul S., Guntawang T., Kochagul V., Photichai K., Sittisak T., Janyamethakul T., Boonprasert K., Khammesri S., Langkaphin W., Punyapornwithaya V. (2019). Possible roles of monocytes/macrophages in response to elephant endotheliotropic herpesvirus (EEHV) infections in Asian elephants (*Elephas maximus*). PLoS ONE.

[23] Guntawang T., Sittisak T., Srivorakul S., Kochagul V., Photichai K., Thitaram C., Sthitmatee N., Hsu W.-L., Pringproa K. (2020). In vivo characterization of target cells for acute elephant endotheliotropic herpesvirus (EEHV) infection in Asian elephants (*Elephas maximus*). Sci. Rep..

[24] Pringproa K., Khonghiran O., Kunanoppadol S., Potha T., Chuammitri P. (2014). In vitro virucidal and virustatic properties of the crude extract of Cynodon dactylon against porcine reproductive and respiratory syndrome virus. Vet. Med. Int..

[25] Gonzalez M., Saiz J., Laor O., Moore D. (1991). Antigenic stability of foot-and-mouth disease virus variants on serial passage in cell culture. J. Virol..

[26] Stanton J.J., Zong J.-C., Latimer E., Tan J., Herron A., Hayward G.S., Ling P.D. (2010). Detection of pathogenic elephant endotheliotropic herpesvirus in routine trunk washes from healthy adult Asian elephants (*Elephas maximus*) by use of a real-time quantitative polymerase chain reaction assay. Am. J. Vet. Res..

[27] Kotila-Row A. (2015). Detection of elephant endotheliotropic herpesvirus (EEHV) in Asian (Elephas maximus) and African elephants (Loxodonta africana). https://stud.epsilon.slu.se/7893/.

[28] Pringproa K., Chungpivat S., Panyathong R., Thanawongnuwech R. (2006). *Culex tritaeniorhynchus* is unlikely to be a vector for the Porcine Reproductive and Respiratory Syndrome virus (PRRSV). Thai J. Vet. Med..

[29] Vitenshtein A., Weisblum Y., Hauka S., Halenius A., Oiknine-Djian E., Tsukerman P., Bauman Y., Bar-On Y., Stern-Ginossar N., Enk J. (2016). CEACAM1-mediated inhibition of virus production. Cell Rep..

[30] Stöckbauer P., Malaskova V., Soucek J., Chudomel V. (1983). Differentiation of human myeloid leukemia cell lines induced by tumor-promoting phorbol ester (TPA). I. Changes of the morphology, cytochemistry and the surface differentiation antigens analyzed with monoclonal antibodies. Neoplasma.

[31] Chanput W., Peters V., Wichers H. (2015). THP-1 and U937 Cells. The Impact of Food Bioactives on Health.

[32] Kabel P.J., De Haan-Meulman M., Voorbij H.A., Kleingeld M., Knol E.F., Drexhage H.A. (1989). Accessory cells with a morphology and marker pattern of dendritic cells can be obtained from elutriator-purified blood monocyte fractions. An enhancing effect of metrizamide in this differentiation. Immunobiology.

[33] Moriuchi H., Moriuchi M., Fauci A.S. (1998). Differentiation of promonocytic U937 subclones into macrophagelike phenotypes regulates a cellular factor (s) which modulates fusion/entry of macrophagetropic human immunodeficiency virus type 1. J. Virol..

[34] Puerta-Guardo H., Mosso C., Medina F., Liprandi F., Ludert J.E., del Angel R.M. (2010). Antibody-dependent enhancement of dengue virus infection in U937 cells requires cholesterol-rich membrane microdomains. J. Gen. Virol..

[35] Tenney D.J., Morahan P.S. (1987). Effects of differentiation of human macrophage-like U937 cells on intrinsic resistance to herpes simplex virus type 1. J. Immunol..

[36] Kabanova A., Marcandalli J., Zhou T., Bianchi S., Baxa U., Tsybovsky Y., Lilleri D., Silacci-Fregni C., Foglierini M., Fernandez-Rodriguez B.M. (2016). Platelet-derived growth factor-α receptor is the cellular receptor for human cytomegalovirus gHgLgO trimer. Nat. Microbiol..

[37] Sinzger C., Jahn G. (1996). Human cytomegalovirus cell tropism and pathogenesis. Intervirology.

[38] Soroceanu L., Akhavan A., Cobbs C.S. (2008). Platelet-derived growth factor-α receptor activation is required for human cytomegalovirus infection. Nature.

[39] Wang X., Huang D.Y., Huong S.-M., Huang E.-S. (2005). Integrin αvβ3 is a coreceptor for human cytomegalovirus. Nat. Med..

[40] De Bruin M., Peters G.J., Oerlemans R., Assaraf Y.G., Masterson A.J., Adema A.D., Dijkmans B.A., Pinedo H.M., Jansen G. (2004). Sulfasalazine down-regulates the expression of the angiogenic factors platelet-derived endothelial cell growth factor/thymidine phosphorylase and interleukin-8 in human monocytic-macrophage THP1 and U937 cells. Mol. Pharmacol..

[41] Savikko J., von Willebrand E. (2001). Coexpression of platelet-derived growth factors AA and BB and their receptors during monocytic differentiation. Transplantation Proceedings.

